# Rapid direct aperture optimization via dose influence matrix based piecewise aperture dose model

**DOI:** 10.1371/journal.pone.0197926

**Published:** 2018-05-23

**Authors:** Xuejiao Zeng, Hao Gao, Xunbin Wei

**Affiliations:** 1 Med-X Research Institute and School of Biomedical Engineering, Shanghai Jiao Tong University, Shanghai, China; 2 School of Biomedical Engineering and Department of Mathematics, Shanghai Jiao Tong University, Shanghai, China; 3 School of Physics and Opto-Electronic Engineering, Foshan University, Foshan, P.R. China; North Shore Long Island Jewish Health System, UNITED STATES

## Abstract

In the traditional two-step procedure used in intensity-modulated radiation therapy, fluence map optimization (FMO) is performed first, followed by use of a leaf sequencing algorithm (LSA). By contrast, direct aperture optimization (DAO) directly optimizes aperture leaf positions and weights. However, dose calculation using the Monte Carlo (MC) method for DAO is often time-consuming. Therefore, a rapid DAO (RDAO) algorithm is proposed that uses a dose influence matrix based piecewise aperture dose model (DIM-PADM). In the proposed RDAO algorithm, dose calculation is based on the dose influence matrix instead of MC. The dose dependence of aperture leafs is modeled as a piecewise function using the DIM. The corresponding DIM-PADM-based DAO problem is solved using a simulated annealing algorithm.The proposed algorithm was validated through application to TG119, prostate, liver, and head and neck (H&N) cases from the common optimization for radiation therapy dataset. Compared with the two-step FMO–LSA procedure, the proposed algorithm resulted in more precise dose conformality in all four cases. Specifically, for the H&N dataset, the cost value for the planned target volume (PTV) was decreased by 32%, whereas the cost value for the two organs at risk (OARs) was decreased by 60% and 92%. Our study of the proposed novel DIM-PADM-based RDAO algorithm makes two main contributions: First, we validate the use of the proposed algorithm, in contrast to the FMO–LSA framework, for direct optimization of aperture leaf positions and show that this method results in more precise dose conformality. Second, we demonstrate that compared to MC, the DIM-PADM-based method significantly reduces the computational time required for DAO.

## Introduction

Intensity modulated radiation therapy (IMRT) [1–4] can produce a high degree of dose conformality directed at tumor targets while sparing the organs at risk (OARs). Traditional IMRT methods involve two steps: fluence map optimization (FMO) [5–7] followed by use of the leaf sequencing algorithm (LSA) [8–10]. Without geometric constraints from multileaf collimators (MLC) taken into consideration, FMO uses beams to solve for fluence maps. LSA then transfers each fluence map [11,12] into a set of deliverable MLC apertures, after which the effective fluence map may be degraded.

By contrast, direct aperture optimization (DAO) [13] bypasses fluence intensity maps and directly solves for deliverable MLC apertures. With regard to optimization algorithms, DAO can be solved using simulated annealing (SA) [2,14,15], genetic algorithms [16,17],gradient-based methods [18], or column generation methods [19,20]. DAO can be modeled on -Monte Carlo (MC) [21] dose calculation; an approximation of MC, such as the pencil beam model; or dose influence matrix (DIM)-based analytical models. Although MC dose calculation is an accurate approach, it is often time-consuming. In a study by Shepard *et al*. [13], the time required for a C-shaped target with seven apertures was approximately 12 minutes when the MC-based DAO method was used. In a study by Cassioli and Unkelbach [22], the dose aperture model was formulated as a piecewise-linear function with respect to aperture leaf positions, and DIM was used so that the gradients could be efficiently computed for local leaf position refinement. In the work of De Gersem *et al*. [23], an analytic dose-difference model was developed according to predefined leaf movements for fast dose calculation. Alternatively, the column generation method models all possible apertures with linear combinations of DIM and iteratively updates the most likely aperture by solving a pricing problem. A DAO method based on the index concept has been in clinical use since 1996. The rapid DAO (RDAO) method proposed in the present paper shortens treatment time and improves treatment accuracy. The proposed method requires fewer apertures than do the other methods, which may be a benefit in the method’s clinical application.

To achieve efficient DAO, we begin by modeling the dose dependence as a function of aperture leaf positions and weights for a given number of apertures, as done similarly in previous DAO studies. The number of optimization variables under consideration is, therefore, relatively small (approximately 100). We then develop a global optimization algorithm based on SA that is efficient for solving medium-scale-or-larger optimization problems. Finally, we utilize a DIM-based piecewise aperture dose model (PADM) for rapid dose calculation.

## Methods

### Objective function

A popular objective function is minimization of the least-squares difference between simulated dose, based on a certain dose aperture model, and prescribed dose.

In this article, we consider the following objective function *F* with respect to the aperture set *A* with a fixed number of apertures indexed by *k*, each of which consists of *m* pairs of aperture leafs that can be fully characterized by three aperture variables—aperture weight *α*, left leaf position *x*, and right leaf position *y*:

$$F(\alpha ,\;x,\;y)=\sum_{s\in S}\frac{w_{s}}{N_{s}}\sum_{i\in V_{s}}{\left(\sum_{(k,m)\in A}\alpha_{k}f_{ikm}(x_{km},y_{km})-d_{i}\right)}^{2}.$$ (1)

We also enforce nonnegative constraints on the aperture weights; thus, the DAO problem can be formulated as

$$\underset{\alpha \geq 0,x,y}{\min}F(\alpha ,x,y).$$ (2)

In Eq (1), *d* is the prescribed dose indexed by *i* and *f*_*ikm*_ is the aperture dose model from the (*k*,*m*)th aperture leaf to the *i*th patient voxel, which is specified next. *S* represents the patient structures, including targets and OARs, and is indexed by *s*, whereas *w*_*s*_ is the weight on the *s*th structure. *N*_*s*_ is the total number of voxels in the *s*th structure, and *V*_*s*_ is the index set for voxels in the *s*th structure. Here *w* indicates the relative significance of various structures to the objective function, and their summation is equal to unity.

### Aperture dose model

**Fig 1:**
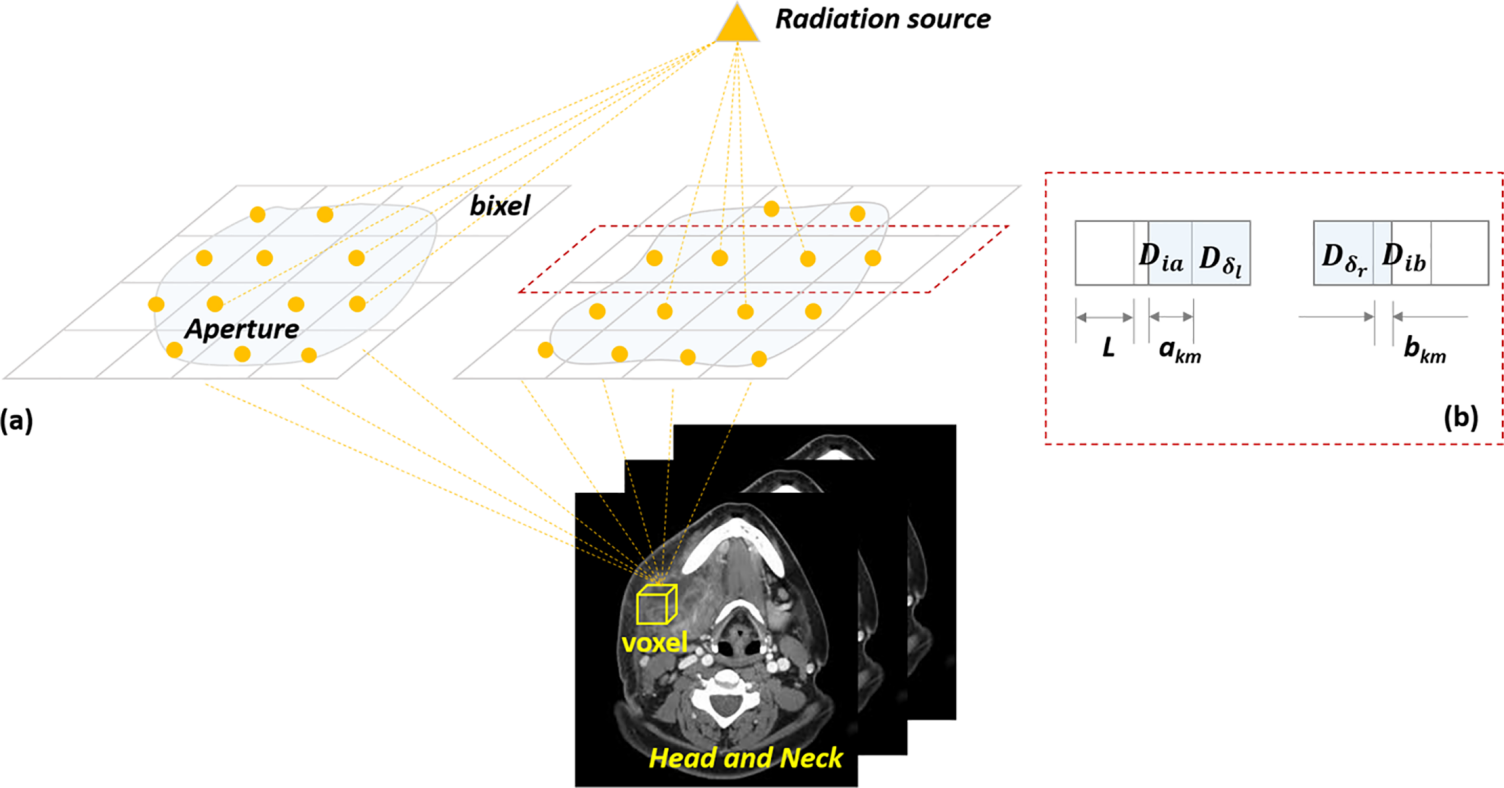
Piecewise-linear aperture dose model. (a) Schematic of radiation. The dose at a specific voxel is a weighted sum of irradiation through each bixel. (b) General scheme with partial bixels: the total dose distribution at the *i*th voxel is the sum of all DIM elements based on piecewise-linear dose calculation.

The motivation to use the DIM for the aperture dose model in Eq (1) is illustrated in Fig 1. Let *D*_*ij*_ be the DIM element denoting the contribution from the *j*th aperture bixel to the *i*th patient voxel. Each aperture leaf pair (*k*,*m*) is divided into a fixed number of aperture bixels of size *L*, and the left and right leaf positions are denoted by (*x*_*km*_,*y*_*km*_). The entire *i*th bixel falling into the opening of the (*k*,*m*)th aperture leaf is formally denoted by *iϵ*(*k*,*m*), and *a*_*km*_ and *b*_*km*_ respectively denote the leftmost and rightmost bixel that fully falls into (*x*_*km*_,*y*_*km*_). For the bixels (leftmost and rightmost) that partially belong to the (*k*,*m*)th aperture leaf, *L*_*lkm*_ or *L*_*rkm*_ is used to represent the length of the area that falls into (*x*_*km*_,*y*_*km*_), and *D*_*ia*_ or *D*_*ib*_ are used for the corresponding DIM element.

As illustrated in Fig 1(A), when the aperture leaf (*x*_*km*_,*y*_*km*_) contains an integer number of bixels (i.e., does not contain partial bixels), the total contribution from this aperture leaf to the *i*th voxel can be regarded as the superposition of the DIM from all the bixels; that is,

$$f_{ikm}(x_{km},y_{km})=\sum_{j\in (x_{km},y_{km})}D_{ij}.$$ (3)

When the aperture leaf contains partial bixels, as shown in Fig 1(B), we model the DIM contribution from partial bixels as proportional to their overlapping length with the aperture leaf; that is,

$$f_{ikm}(x_{km},y_{km})=D_{ia}\frac{a_{km}}{L}+\sum_{j\in (x_{km},y_{km})}D_{ij}+D_{ib}\frac{b_{km}}{L},$$ (4)

where each bixel is assumed to have the same length *L*. *a*_*km*_ and *b*_*km*_ qualify the partial opened length where the left and right leaf posit and *δ*_*l*_ = ceil(*x*_*km*_) and *δ*_*r*_ = floor(*y*_*km*_).

Eq (4) represents a DIM-PADM. Note that high-order polynomials can be used in the DIM-PADM instead of the piecewise-linear model shown in Eq (4). Because the DIM-PADM has an explicit form based on the DIM, it is solved much faster than the MC model and therefore may be used in an RDAO algorithm.

### Optimization algorithm

**Fig 2:**
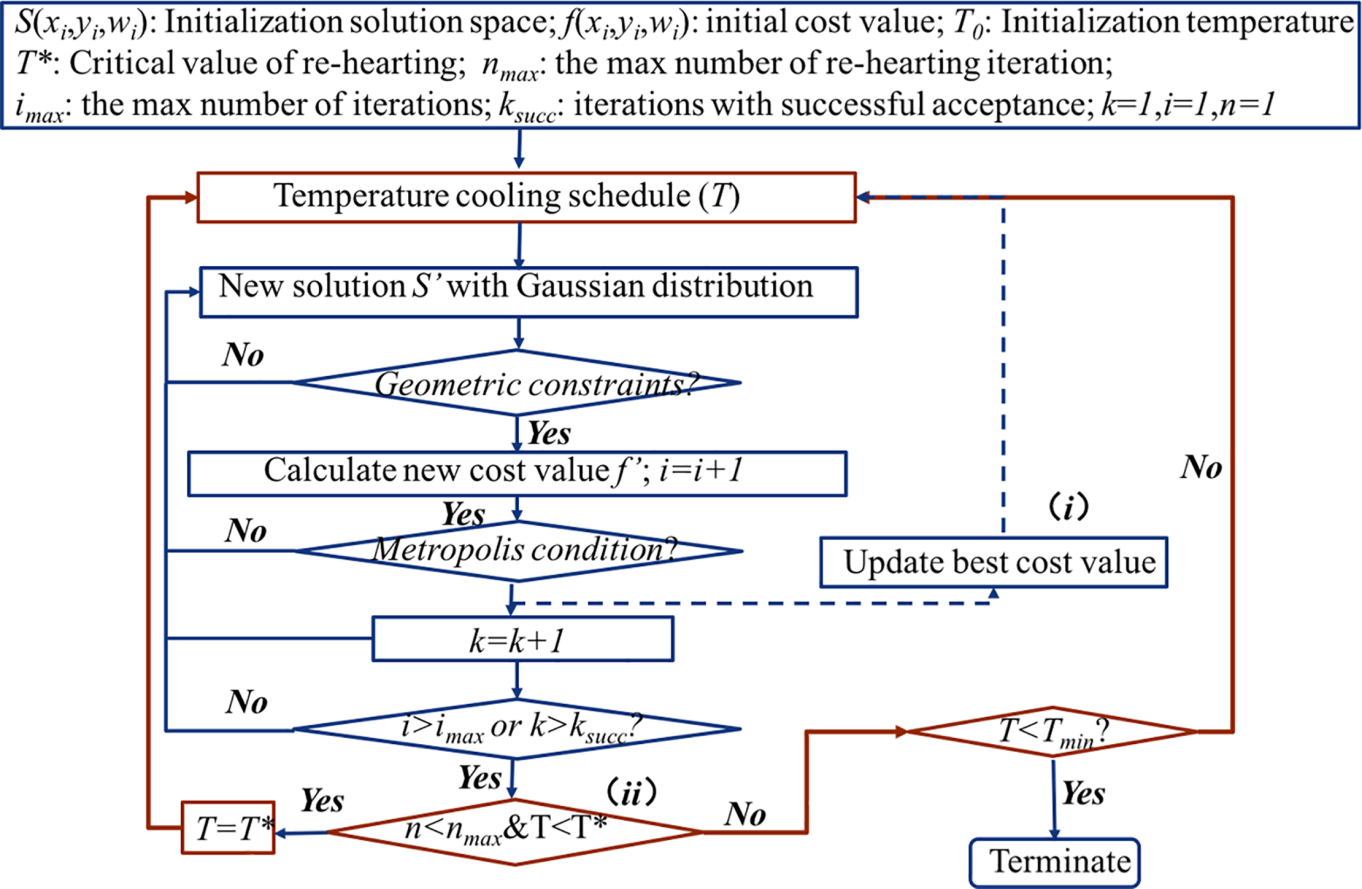
SA algorithm for rapid DAO.

A modified SA algorithm is used to solve the DIM-PADM-based DAO problem (Eq (2)). The key feature of the SA algorithm is that it can escape local optima with mechanism (i.e., accept the worse cost value) in contrast to other optimization methods such as the greedy algorithm. Moreover, SA enables the inclusion of constraint conditions, such as aperture constraints and dose normalization, and is therefore more useful than a developed software package. We make two modifications to the traditional SA algorithm: we iteratively update the current best solution ((*i*) in Fig 2), and we extend the search area ((*ii*) in Fig 2). The SA algorithm is based on simulation of the minimum temperature reached during a solid substance’s cooling process. Therefore, in an SA algorithm, the minimization function is analogous to the change in a specific system from an arbitrary state to a steady state with the lowest possible internal energy.

As in shown in Fig 2, inputs are assigned both randomly and logically. In the inner iteration loop (blue lines in Fig 2), the temperature is constant and we stochastically sample a Gaussian distribution. We then add the Gaussian distribution into optimization variables during each iteration. The mean of the Gaussian distribution is zero and its width decreases according to the following [14,24]:

$$\sigma =1+\left(L_{0}‑1\right)e^{\log(k_{succ}+1)\alpha }$$ (5)

where *L*_*0*_ is the initial width and *α* donates the cooling schedule. The variables are updated if the corresponding Gaussian distribution satisfies the constraints, such as those of the interval between the left and right leafs; otherwise the Gaussian distribution is re-sampled (condition (1)).

The metropolis criterion is used to determine whether to accept the solution to a new state. If the value of the objective function has decreased, the probability of acceptance equals 1. Otherwise, the probability is given by the standard Boltzmann SA cooling schedule [25–27]. Additionally, a random float point probability within [0,1] is generated during each iteration. The probability of acceptance is compared with this random probability to determine whether the new state solution is accepted.

$$P=\{\begin{array}{ll} 1, & F_{i+1}(\alpha ,x,y)<F_{i}(\alpha ,x,y)\\ 2P_{0}\frac{1}{1+e^{\log(k_{succ}+1)/\beta }}, & otherwise \end{array}$$ (6)

where *P*_*0*_ denotes the initial probability and *β* denotes the rate of cooling. To terminate the SA effectively, we adopt a dual threshold strategy to reduce the computational complexity. The stopping criteria *k*_*succ*_ and *i*_*max*_ are the number of iterations with successful acceptance and maximum number of iterations, respectively. The inner iteration terminates when either *k* equals *k*_*succ*_ or when *i* equals *i*_*max*_. To avoid missing the current optimal solution, the improvement algorithm increases the memory function to remember the current best state; thus, the modified SA algorithm is intelligent.

In the outer loop (red lines in Fig 2), we extend the search area by reraising the temperature while the temperature is less than T*, which is the threshold used for several iterations. In this process, *T*_*min*_ is the termination temperature. The improvement algorithm can re-search the area while the iteration is in progress, thus increasing the possibility of finding a better solution. Finally, the algorithm terminates when the temperature becomes less than *T*_*min*_.

## Materials and results

The common optimization for radiation therapy (CORT) dataset [28] was used as benchmark with which to validate the proposed RDAO algorithm in comparison with a two-step FMO–LSA algorithm. In the comparative experiments, the same IMRT beam angles and apertures were used for RDAO and FMO–LSA. In all the experiments, the FMO-LSA method has reached the optimization values. For FMO–LSA, FMO solved the weighted least-square objective function, similar to Eq (1), with nonnegative constraints on fluence maps, by using quadratic programming; LSA was performed according to the method proposed by Engel [29]. The CORT dataset included four case types: TG119, prostate, liver, and head and neck (H&N) cases. In the forward modeling stage of the present study, we used a DIM-PADM as the dose calculation method (Eq (4)). The DIMs for the four case types were obtained from the open-source CORT dataset. Thus, we were able to compute dose distributions according to arbitrary aperture positions and aperture weights. In the inverse problem stage, we used a modified SA algorithm to optimize aperture positions and weights. That is, we iteratively updated the aperture weights and leaf positions. The purpose of the optimization process was to minimize the objective function shown in Eqs (1) and (2). In the present study, the prescribed dose to the planned target volume (PTV) was 1 and to OARs was 0. At the end of optimization, a final dose calculation was performed using the optimized apertures and aperture weights. The 3D dose distributions were created using the computational environment for radiotherapy research toolbox. We normalize the target dose into the same dose level in our comparative study. Here, we let 95% of the volume receiving the prescribed dose, i.e. 1.

### TG119 case

**Fig 3:**
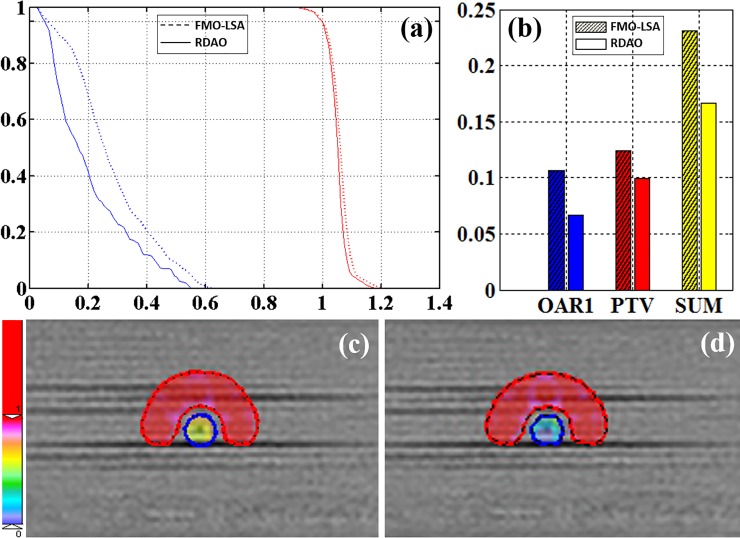
TG119 results. (a) DVH and (b) cost values (red: PTV; blue: OAR; dotted line: FMO–LSA; solid line: RDAO); dose distribution from (c) FMO–LSA and (d) RDAO.

For the TG119 case, we used five beams at gantry angles 0°, 72°, 144°, 216°, and 288°. The apertures at each beam angle were 25, 5, 14, 14, and 7, respectively. The region of interest contained one PTV (OuterTarget) and one OAR (core) with *w*_*PTV*_ = 0.9 and *w*_*OAR*_ = 0.1. As shown in Fig 3, the dose volume histogram (DVH) suggests that RDAO resulted in better protection of the OAR (Fig 3(A)) and a lower cost value than FMO–LSA (Fig 3(B)). The dose distribution results indicate a significant dose conformality improvement occurred with RDAO (Fig 3(C) and 3(D)).

### Prostate case

**Fig 4:**
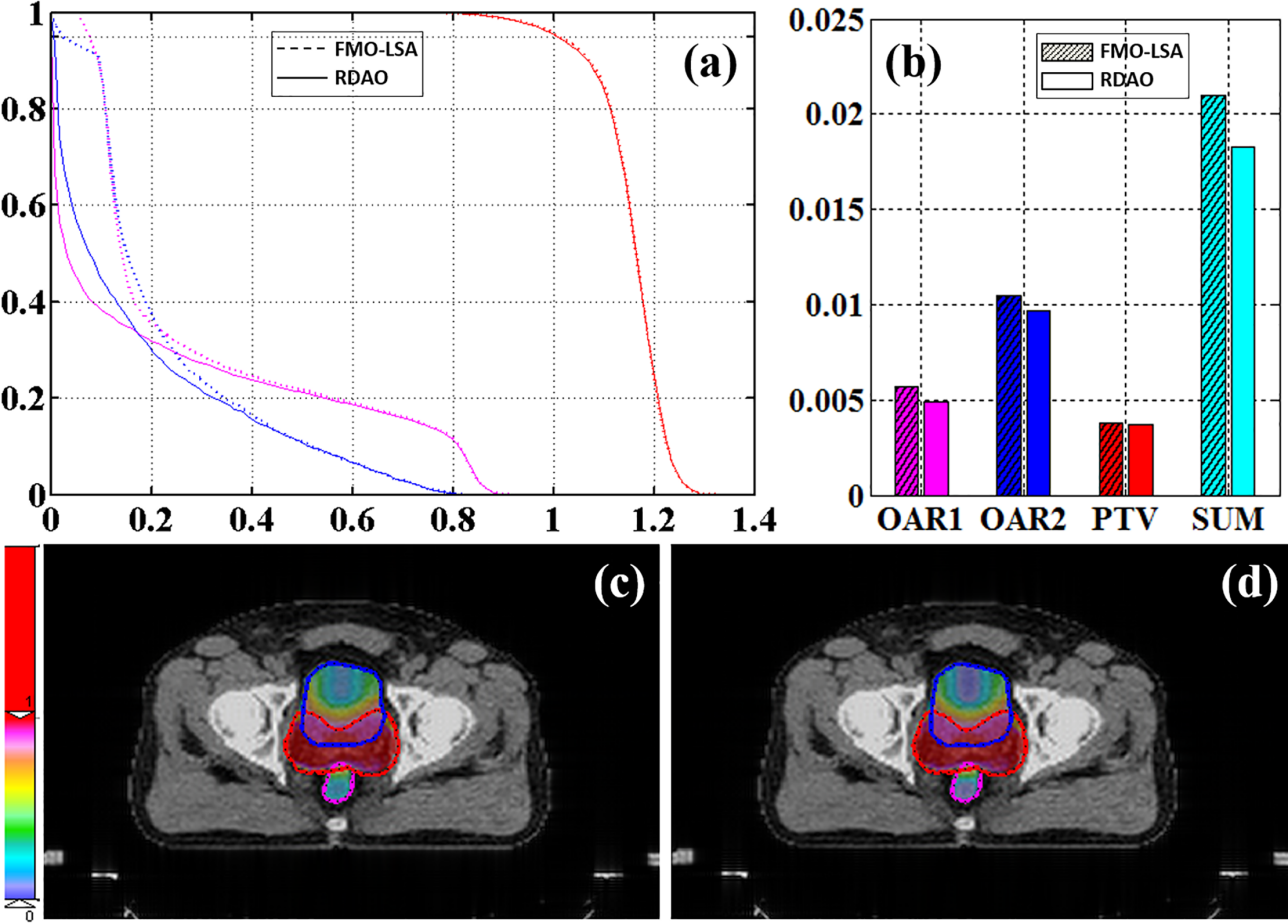
Prostate results. (a) DVH and (b) cost values (red: PTV; magenta: OAR1; blue: OAR2; dotted line: FMO–LSA; solid line: RDAO); dose distribution from (c) FMO–LSA and (d) RDAO.

For the prostate case, 19, 13, 14, 16, and 14 apertures were applied to five coplanar beams at 0°, 72°, 144°, 216°, and 288° gantry angles. The region of interest contained one PTV (PTV_68) and two OARs (OAR1: rectum and OAR2: bladder), with *w*_*PTV*_ = 0.9, *w*_*OAR1*_ = 0.05, and *w*_*OAR2*_ = 0.05. For the prostate dataset (Fig 4), RDAO resulted in significantly higher protection of the OARs than FMO–LSA, especially for irradiation near 0, as indicated by the decrease in the cost value (Fig 4(B)) and decreases in the DVH (Fig 4(A)). Although the improvement in the DVH was nonsignificant (Fig 4(A)), both components of the cost value decreased for the PTV.

### Liver case

**Fig 5:**
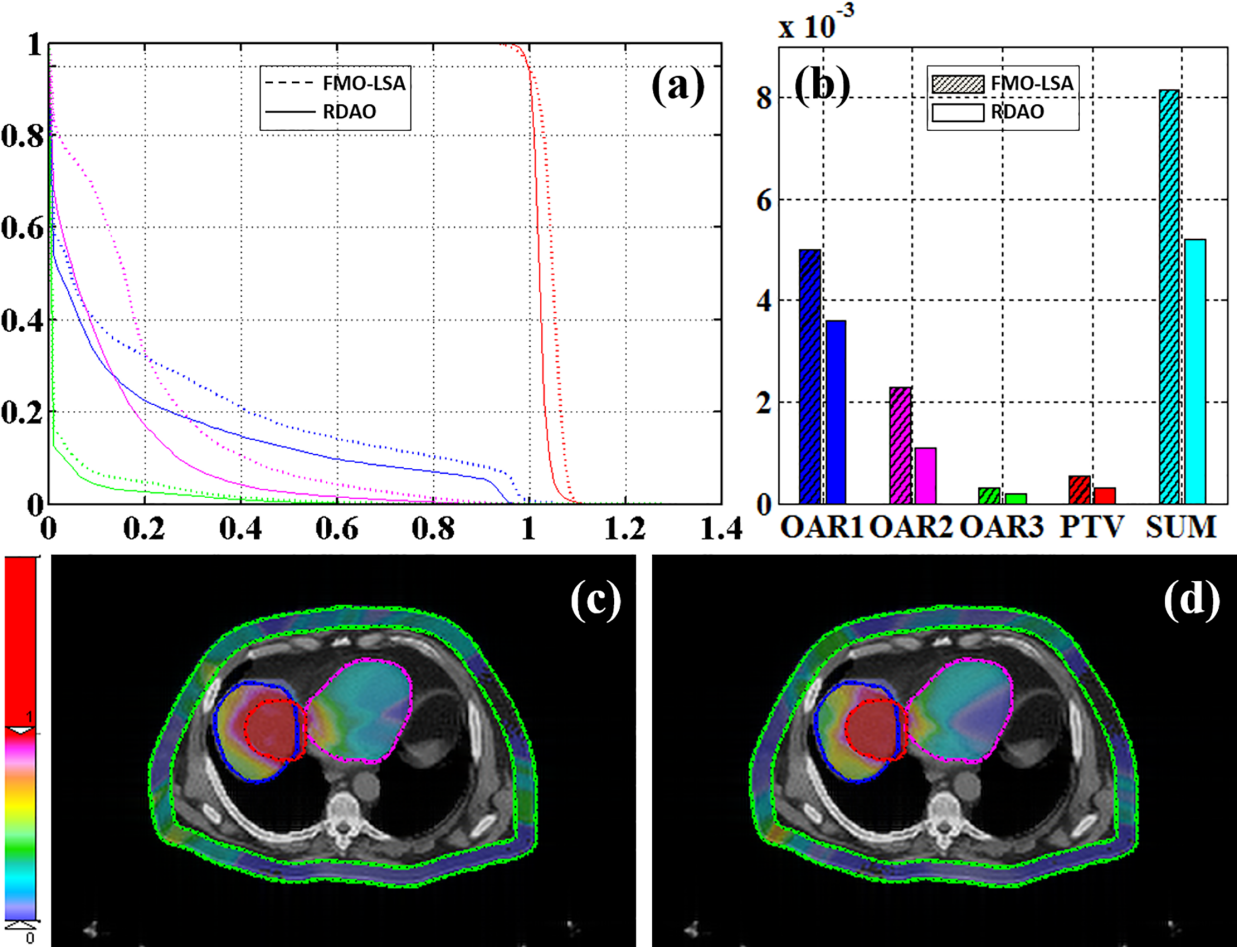
Liver results. (a) DVH and (b) cost values (red: PTV; blue: OAR1; magenta: OAR2; green: OAR3; dotted line: FMO–LSA; solid line: RDAO); dose distribution from (c) FMO–LSA and (d) RDAO.

For the liver case, we utilized 6, 8, 9, 10, 10, 7, and 11 apertures for seven noncoplanar beams at (gantry, couch) angles (58°, 0°), (106°, 0°), (212°, 0°), (328°, 0°), (216°, 32°), (226°, −13°) and (296°, 17°), respectively. The region of interest contained one PTV and three OARs (OAR1: liver, OAR2: heart, and OAR3: entrance) with *w*_*PTV*_ = 0.9, *w*_*OAR1*_ = *0*.1/3, *w*_*OAR2*_ = 0.1/3, and *w*_*OAR3*_ = 0.1/3. For the liver dataset (Fig 5), a prominent performance boost can be observed in DVH, cost value, and the dose distribution map. The cost values for the respective OARs decreased by 28%, 52%, and 36%, and for the PTV by 40% (Fig 5(B)).

### H&N

**Fig 6:**
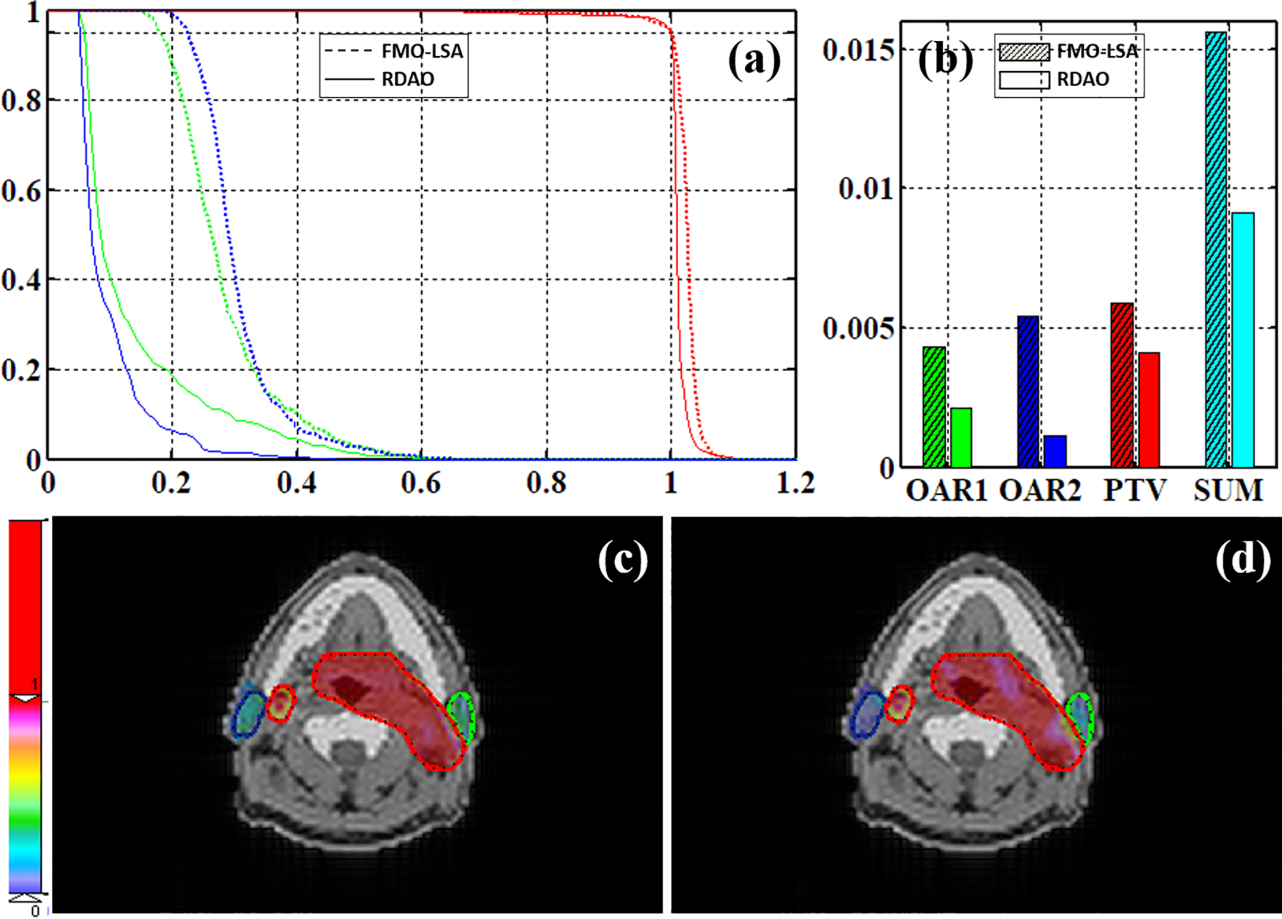
H&N results. (a) DVH and (b) cost values (red: PTV; blue: OAR1; green: OAR2; dotted line: FMO–LSA; solid line: RDAO); dose distribution from (c) FMO–LSA and (d) RDAO.

In the H&N case, 10 gantry angles were used with five beams at couch = 0°(0°, 72°, 144°, 216°, 288°) and five beams at couch = 20°(180°, 220°, 260°, 300°, 340°). In total, 10 noncoplanar beams with 10 apertures for each beam were thus used. The region of interest contained one PTV (PTV_70) and two OARs (OAR1: left parotid and OAR2: right parotid) with *w*_*PTV*_ = 0.8, *w*_*OAR1*_ = 0.1, and *w*_*OAR2*_ = 0.1. Compared with the results of using FMO-LSA, use of the proposed method decreased the cost values of OAR1, OAR2, and PTV by 60%, 92%, and 32%, respectively (Fig 6(B)). The DVH shows that RDAO was more protective than FMO–LSA for the left parotid and right parotid. As displayed in Fig 6(C) and Fig 6(D), compared with the corresponding FMO–LSA map, the PTV dose distribution map of RDAO is more homogeneous and has a dose scale closer to 1. The corresponding FMO–LSA map shows local heterogeneous areas.

From these four cases, the resulting DVH, global cost value, and dose distribution indicate that use of the RDAO method led to more precise dose conformality than use of the traditional two-step FMO–LSA method. Moreover, the proposed method also decreased the cost value for each OAR. For instance, in the liver case, the cost value for the three OARs was 28%, 52%, and 36% lower and that for the PTV was 40% lower, respectively than those obtained using the FMO–LSA method. MC-method-based DAO is accurate but is limited by the long time required for simulated sampling. In this study, we proved that the proposed DIM-PADM-based DAO method is a fast and accurate method for IMRT. For example, in the liver case, the computation time for dose calculation based on DIM-PADM was approximately 0.01 s, and total time taken to optimize the treatment plan was less than 3 minutes. By contrast, the MC method has been shown to require more than 25 minutes with the same configurations (seven apertures, Core i7-4930K CPU, and 16 GB RAM).

## Discussion

Compared with conventional sequencing method, e.g. Engle method after FMO, the proposed RDAO method can produce higher dose conformality. In the proposed method, aperture leaves are allowed to move between the bixels. This improvement in precision not only increases dose conformality, it also allows the reduction in the number of apertures per beam angle to perform the required quality assurance procedures.

Another advantage of the proposed algorithm is that the RDAO method works much faster than MC-based DAO. MC-based DAO method is limited by its long sampling time. The proposed RDAO method adopts SA algorithm with quick convergence to achieve the optimal solution. However, the optimal solutions are related to SA parameters and may fall into the local optimal solution. In our work, we increase the possibility to find the optimal solution by re-raising the temperature. We will continue to work on the improvements of the global optimization algorithm in the future work.

## Conclusions

Here we propose a novel RDAO method for efficient IMRT. The RDAO algorithm uses a DIM-PADM to calculate doses and an improved SA algorithm to optimize the dose distribution. Results show that RDAO achieves higher dose conformality and requires fewer apertures per beam angle than traditional sequencing method, e.g. Engle method. Moreover, the proposed method is much faster than MC-method-based DAO. This method improves both the accuracy and efficiency of IMRT inverse planning.
